# Proteomics-Based Identification of Retinal Protein Networks Impacted by Elevated Intraocular Pressure in the Hypertonic Saline Injection Model of Experimental Glaucoma

**DOI:** 10.3390/ijms241612592

**Published:** 2023-08-09

**Authors:** Khadiza Zaman, Vien Nguyen, Katalin Prokai-Tatrai, Laszlo Prokai

**Affiliations:** Department of Pharmacology and Neuroscience, University of North Texas Health Science Center, Fort Worth, TX 76107, USA; khadiza.zaman@unthsc.edu (K.Z.); vien.nguyen@unthsc.edu (V.N.)

**Keywords:** retina proteomics, glaucoma, ocular hypertension, Brown Norway rat model, bioinformatics, pathway analysis, protein networks

## Abstract

Elevated intraocular pressure is considered a major cause of glaucomatous retinal neurodegeneration. To facilitate a better understanding of the underlying molecular processes and mechanisms, we report a study focusing on alterations of the retina proteome by induced ocular hypertension in a rat model of the disease. Glaucomatous processes were modeled through sclerosing the aqueous outflow routes of the eyes by hypertonic saline injections into an episcleral vein. Mass spectrometry-based quantitative retina proteomics using a label-free shotgun methodology identified over 200 proteins significantly affected by ocular hypertension. Various facets of glaucomatous pathophysiology were revealed through the organization of the findings into protein interaction networks and by pathway analyses. Concentrating on retinal neurodegeneration as a characteristic process of the disease, elevated intraocular pressure-induced alterations in the expression of selected proteins were verified by targeted proteomics based on nanoflow liquid chromatography coupled with nano-electrospray ionization tandem mass spectrometry using the parallel reaction monitoring method of data acquisition. Acquired raw data are shared through deposition to the ProteomeXchange Consortium (PXD042729), making a retina proteomics dataset on the selected animal model of glaucoma available for the first time.

## 1. Introduction

Glaucoma is the second leading cause of blindness worldwide and describes a group of ocular disorders with multi-factorial etiology united by a clinically characteristic optic neuropathy [1,2]. During the disease, retinal ganglion cells (RGCs) and their axons forming the optic nerve are gradually destroyed, which leads to diminishing vision [3,4,5,6]. Open-angle glaucoma is the most common form, whose prevalence increases with aging [7,8] and elevated intraocular pressure (IOP) [6], manifesting as ocular hypertension (OHT) [9]. However, mechanisms of glaucomatous neurodegeneration due to OHT have been poorly understood. While recent advances in mass spectrometry (MS)-based retina proteomics have been promising a better understanding of various retinal neuropathies [10], only a few proteomics studies have been published thus far with a focus on the glaucomatous retinae. Most of them analyzed tissues obtained from animal models of glaucoma, although the proteome of the diseased versus healthy human retinae has also been compared [11,12].

Using rodent models to gain insights into glaucomatous retinopathy, early investigations utilized gel electrophoresis to detect differentially expressed proteins for subsequent MS-based identification [13,14,15,16], a methodology that has limitations in throughput and proteome coverage [17,18]. To address these shortcomings, gel-free retina shotgun proteomics was utilized in a nonhuman primate (NHP) study relying on unilateral optic nerve transection to produce early experimental glaucoma [19]. Nevertheless, proteome coverage in this effort was still inadequate and afforded few tangible mechanisms tied to the underlying retinopathy. Specifically, the study concluded that proteomic changes were condition-specific, involving cytoarchitecture regulation as an early response to chronic IOP elevation. Owing to high costs, practical constraints, and limited accessibility to most researchers in the field [20], there have been no subsequent retina proteomics investigations involving NHP models of glaucoma. On the other hand, rodent hypertensive glaucoma models have been the preferred options to understand molecular processes, mechanisms, and structural changes associated with the development and progression of the disease [21,22]. For proteomics-based interpretation of the glaucomatous process, two large-scale studies using rats as experimental species have been reported so far [23,24]. 

In the first of these proteomics studies [23] that used cauterization of episcleral veins to increase IOP as a glaucoma model in female Sprague-Dawley rats [25], extracted retina proteins were fractionated by sodium dodecyl sulfate–polyacrylamide gel electrophoresis (SDS–PAGE) for subsequent data-dependent liquid chromatography–electrospray ionization tandem mass spectrometry (LC–ESI-MS/MS) analyses. Out of 961 identified and quantified retina proteins, 32 met the authors’ inclusion criteria of a minimum two-fold change in expression to consider the protein upregulated or downregulated besides showing statistically significant differences in the measured abundances between the OHT and normotensive groups. Retina proteins downregulated and upregulated by the elevated IOP based on the employed label-free quantification (LFQ) method (16 proteins each) were functionally associated mainly with cell differentiation, apoptosis, and stress response.

The second study involving ocular microbead injections to induce OHT [26] used a multiplexed bottom-up proteomics approach based on labeling with stable-isotope coded tandem mass tags (TMT) and synchronous precursor selection mass spectrometry (SPS-MS3) after the online separation of tryptic peptides by nanoflow liquid chromatography (nLC) [24]. With a fold change of ≤0.83 as the threshold for downregulation, elevated IOP was found to reduce the expression of 139 proteins associated, among others, with glutathione metabolism, mitochondrial dysfunction/oxidative phosphorylation, cytoskeleton, and actin filament organization. Increased expressions were found for 109 proteins when a fold change of ≥1.2 was used as the threshold for upregulation, for which the coagulation cascade, apoptosis, oxidative stress, and RNA processing were the major overrepresented processes. OHT-induced differential modulation of nuclear receptor signaling, cellular survival, protein synthesis, transport, and cellular assembly pathways were implicated through functional network analyses from the findings. Comparisons of OHT-induced changes in protein expressions observed in this study with results of a previous retina proteomics study of glaucomatous versus healthy human samples [12] revealed that downregulation of crystallins and glutathione-metabolizing enzymes, as well as mitochondrial dysfunction, were the major common processes. However, the intraocular microbead injection model of glaucoma in rats [26] did not replicate all features of proteome alteration associated with the human disease. For example, the activation of the complement pathway and upregulation of cholesterol transport-related proteins were unique observations for glaucomatous human retinae. Unfortunately, raw data of the proteomics studies on induced OHT models of glaucoma in rats and human glaucomatous retinae were not shared with the scientific community, which precludes their comparative reanalyses.

Here, we report an LFQ-based retina proteomics study involving the hyperosmolar saline injection model of glaucoma in rats [27,28]. This model has been usually performed using Brown Norway (BN) rats and with male retired breeders in most cases [29]. Partly owing to the superior spatial acuity of their vision, this strain is more frequently used in glaucoma research than the albino (e.g., Sprague–Dawley) strains [30]. Acquired raw data were shared through deposition to the ProteomeXchange Consortium [31] by the PRIDE partner repository (assigned dataset identifier: PXD042729), making a complete retina proteomics dataset on an induced OHT model of glaucoma available for the first time. 

## 2. Results

Using data-dependent nLC–MS/MS for discovery-driven assessment of the normotensive and OHT retina proteome in retired breeder male BN rats, Proteome Discoverer running the Mascot search engine and relying on the Uniprot rat protein database returned 4424 high-confidence protein identifications with at least two identified proteotypic peptides (Table S1 of the Supporting Information available online). More than 1100 proteins were validated subsequently by Scaffold (Table S2), which estimated a false discovery rate (FDR) of 0.1% at the protein level and 0.0% at the peptide level based on rigorous identification criteria and using a decoy-based method of FDR estimation. Our LFQ applied spectral counting and Fisher’s exact tests [32] with corrections for multiple testing [33] to obtain proteins with statistically significant differences in their expression in the OHT versus normotensive retinae (Table S3). Altogether, the elevated IOP affected 204 retina proteins when we filtered the list of these proteins with thresholds of ≤0.5 (downregulation) and ≥2.0 (upregulation) in spectral count ratios. Specifically, 92 proteins were downregulated, and 112 were upregulated in the OHT retinae using these criteria.

Table 1a summarizes the top molecular and cellular processes, while Table 1b lists the top diseases and disorders affected by elevated IOP in the retina proteome. Proteins impacted by the induced OHT were organized into interaction networks by IPA^®,^ as shown in Figure 1 (example) and Figures S1–S10, as well as summarized in Table S4.

**Figure 1:**
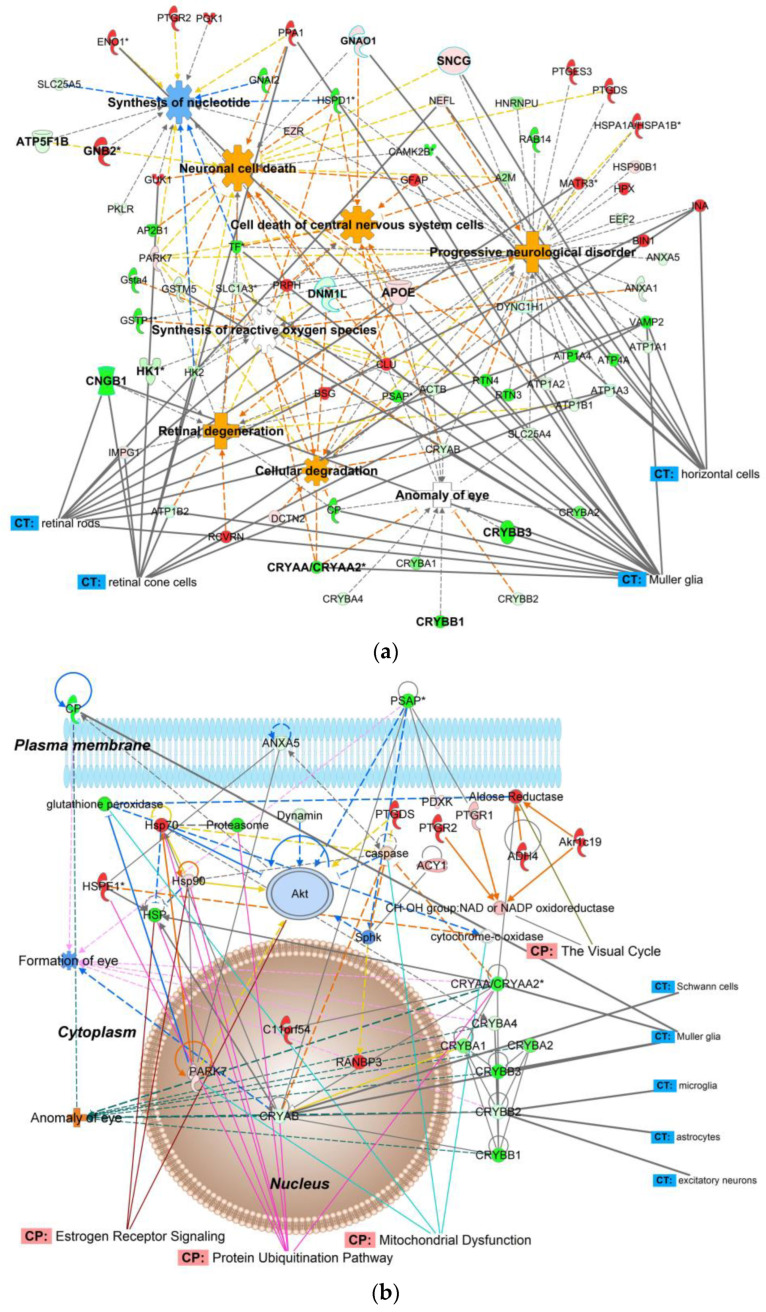
IPA^®^ mapping of OHT-impacted proteins in the aging male BN rat retina. (**a**) A subset of these proteins was mapped to a network associated by the knowledge base with neuronal or CNS cell death, progressive neurological disorder, retinal degeneration, cellular degradation, and anomaly of the eye, as well as nucleotide synthesis and the synthesis of reactive oxygen species. (**b**) An IPA^®^ protein interaction network linked to development disorder, ophthalmic disease, organismal injury, and cellular compromise. Canonical pathways (CP), and cells and tissue (CT) were displayed by the software’s overlay function. Green symbol–downregulation; red symbol–upregulation; blue symbol and line–inhibition/decrease; orange symbol and line–activation/increase; yellow line–activity cannot be predicted; solid line–direct relationship; dashed line–indirect relationship; *—isoforms. Abbreviations of proteins are listed in Table S6.

Along with their regulation patterns, Figure 1a pinpoints proteins that contributed to various pathological processes in the glaucomatous OHT retina. The molecular activity predictor (MAP) tool of IPA^®^ predicted increased retinal degeneration, cellular degradation, and neuronal cell death. Inhibition of nucleotide synthesis and synthesis of reactive oxygen species were among the prominent consequences of IOP elevation. In addition, “anomaly of the eye” was implicated mainly due to the downregulation of crystallins.

Bioinformatics also revealed over 100 canonical pathways that responded to IOP elevation (Table S5). In Figure 1b showing a representative protein interaction network, we used the overlay option of IPA^®^ to point out some canonical pathways impacted by OHT. Specifically, they included mitochondrial dysfunction, estrogen receptor signaling, and the visual cycle. The protein interaction networks shown in Figure 1b and Figures S1–S10 of the Supplementary Materials also label, along with RGCs, other cell types that may be involved in glaucomatous neurodegeneration and could be distinguished by bioinformatic analyses based on the results of our proteomics study. Specifically, the networks indicate protein interactions involving various types of retinal cells (e.g., horizontal cells, photoreceptors, rod and cone cells, Müller glia cells, astrocytes, as well as excitatory, inhibitory, and sensory neurons).

**Figure 2:**
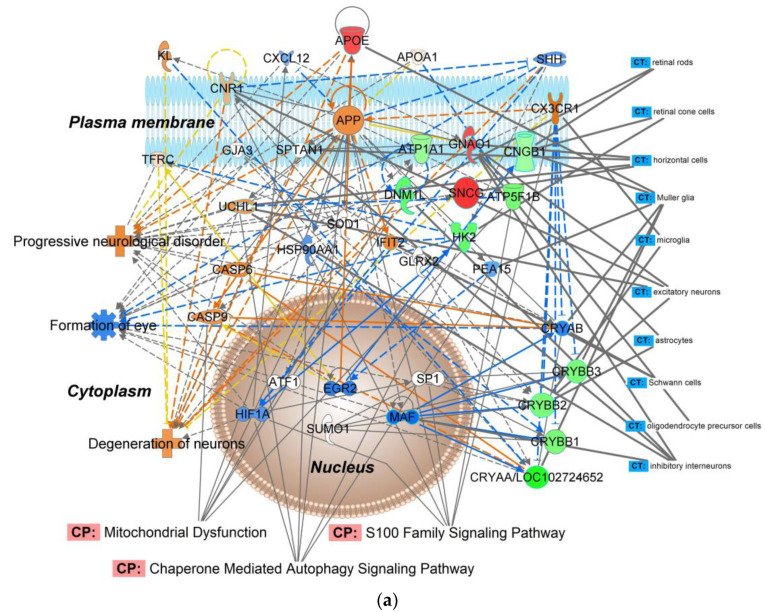

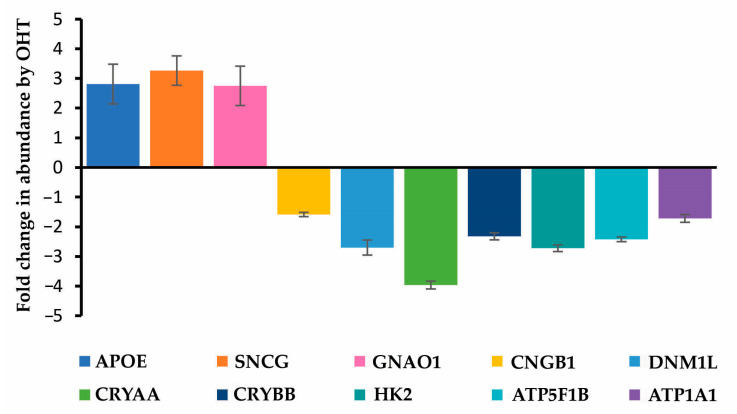
Targeted proteomics-based validation of the selected OHT-impacted male rat retina proteins as biomarkers: APOE, ATP1A1, ATP5F1B, CNGB1, CRYAA, CRYBB, DNM1L, GNAO1, HK2, and SNCG. (**a**) Molecular interaction network generated by IPA^®^ from the selected proteins. IPA^®^’s knowledge base associated this network with cell death and survival, ophthalmic disease, nervous system development and function, organismal injury, and abnormalities. Top diseases, canonical pathways (CP), and cells and tissue (CT) were displayed by the software’s overlay function. Green symbol–downregulation; red symbol–upregulation; blue symbol and line–inhibition/decrease; orange symbol and line–activation/increase; yellow line–activity cannot be predicted; solid line–direct relationship; dashed line–indirect relationship. (**b**) OHT-induced fold changes in the selected proteins obtained by nLC–ESI-MS/MS-based targeted proteomics using the PRM method. Abbreviations of proteins involved in the displayed IPA^®^ network (**a**) are listed in Table S7.

A subset of OHT-impacted proteins listed in Table S3 was chosen for targeted proteomics. The heuristic selection of target proteins aspired to achieve overlap in functions and canonical pathways among the interaction networks established through our discovery-driven study focusing on the effect of the IOP elevation in the chosen experimental glaucoma model (Figure 1b and Figures S1–S10). The IPA^®^ network assembled from the selected ten proteins (apolipoprotein E, APOE; Na^+^/K^+^-transporting ATPase subunit alpha-1, ATP1A1; ATP synthase F1 β-subunit, ATP5F1B; cyclic nucleotide-gated channel subunit β 1, CNGB1; α-crystallin A chain, CRYAA; β-crystallins including β-crystallin B1, CRYBB1, β-crystallin B2, CRYBB2, and β-crystallin B3, CRYBB3, as well as dynamin-1-like protein, DNM1L; guanine nucleotide-binding protein G(o) α-subunit, GNAO1; hexokinase 2, HK2; and γ-synuclein, SNCG) is shown in Figure 2a, which captured the association of the induced OHT broadly with cell death and survival, neurological disease, ophthalmic disease, organismal injury and abnormalities. Top canonical pathways included mitochondrial dysfunction, S100 protein family signaling, and chaperone-mediated autophagy signaling. Figure 2b summarizes results obtained by LC–ESI-MS/MS-based targeted proteomics using the parallel reaction monitoring (PRM) method [34].

## 3. Discussion

In hypertensive glaucoma, the gradual loss of RGCs and damage to the entire visual pathway occurs due to elevated IOP [3,5]. Obstacles to conducting mechanistic studies on glaucoma using clinical specimens have inspired the development of several animal models that replicate features of human glaucoma [20,21,22]. Besides facilitating investigations aimed at understanding the pathophysiology of the disease, these models can also support the discovery of promising protective agents and strategies against glaucomatous neurodegeneration [35,36]. Widely used hypertensive paradigms in rodents involve episcleral vein cauterization [25], obstruction of the trabecular meshwork by injection of microparticles [26], and hypertonic saline injections into the episcleral veins or limbal vessels [27]. Like all models, they have advantages and limitations [21]. However, applying new methodologies, such as retina proteomics, to experimental glaucoma(s) will be of immense value in translational contexts [37,38].

Thus far, only two large-scale whole retina proteomics studies have been reported using induced OHT models of experimental glaucoma in rodents and with a focus on retinal neurodegeneration [23,24]. Besides the species and strain (Sprague-Dawley rats), there was little overlap between the two investigations. Specifically, the first study [23] employed the episcleral vein cauterization model to female animals and SDS-PAGE fractionation followed by conventional data-dependent nLC–ESI-MS/MS analyses processed by the Andromeda search engine and the LFQ method of the MaxQuant software (version 1.5.3.30, Max-Planck Institute for Biochemistry, Martinsried, Germany) [38] to identify retina proteins significantly affected by OHT. The other study [24] applied the microparticle injection model to male rats and relied on stable-isotope-based multiplex TMT-labeling and data-dependent nLC–ESI-SPS-MS3 acquisitions in their proteomics approach. Data processing relied on the Sequest search algorithm [39] and TMT reporter ion-based quantifications [40] to identify retina proteins differentially expressed between the elevated IOP and normotensive control conditions.

Mirzaei et al. [24] reported over 200 retina proteins affected by elevated IOP, but such high numbers were achieved by decreasing the fold change threshold drastically compared to the twofold change in expression applied to the retina proteomics study by Anders et al. [23] to accept the respective proteins up- and downregulated by the induced OHT of their animal model. Although there has been no consensus about thresholding in discovery-driven proteomics, overly relaxed thresholds to boost sheer numbers erode confidence in results, especially considering that the SPS-MS3 approach [40] used by Mirzaei et al. [24] was intended to remedy “ratio compressions” associated with isobaric labeling-based multiplexed quantifications [41]. When one counted retina proteins whose abundance was reported to be changed at least twofold by the elevated IOP, the two studies identified similar numbers of significantly affected retina proteins (32 [23] and 38 [24], respectively). Since raw data from these studies were not made available in public data repositories, rigorous comparative (re)analyses in the context of OHT-induced experimental glaucoma in rats is not possible. Nevertheless, a profound downregulation of crystallins by the elevated IOP in the retina proteome was a common finding both in the episcleral vein cauterization [23] and in the microparticle injection model [24].

Reliance on the albino Sprague–Dawley strain could have been another potential confounding factor in these hypertensive glaucoma-focused retina proteomics studies [23,24]. Pigmented strains such as Brown Norway rats have been found to respond more sensitively to IOP elevation than albino rats [42]. In addition, the hypertonic saline injection method to produce OHT by aqueous outflow obstruction has demonstrated strengths in replicating human glaucoma over alternative paradigms such as episcleral vein cauterization [29,43]. The use of males for the studies initially is also justified because glaucomatous male rats experience higher structural loss and display worse neuroretinal functionality than females [44].

Our discovery-driven proteomics study now presents over 200 proteins significantly affected by IOP elevation in the hypertonic saline injection model applied to male retired breeder Brown Norway rats (Table S3). The results have proven our hypothesis about the benefits of the paradigm for investigating OHT-induced changes in the whole retina proteome compared to previous studies that relied on different glaucoma models and rat strain [23,24], as well as different sex of the experimental animals [23]. Bioinformatics by IPA^®^ on these proteins revealed multitudes of information on the molecular, cellular, and physiological processes in the glaucomatous retinae when compared to the normotensive tissue (Table 1 and Figure 1a). Among the many IPA^®^ networks assembled, the one shown in Figure 1b is particularly relevant to ophthalmic diseases and disorders, capturing many molecular crosstalks that could orchestrate the progression of OHT-induced neurodegeneration in the retina, including those associated with glaucomatous processes. The unfavorable downregulation of α- and β-crystallins in the hypertensive retina has been observed in other proteomics studies [23,24] and could indicate vulnerability toward degenerative processes. Since the pathophysiology of glaucoma is not well understood [4], many additional associations involving OHT-impacted proteins identified from our experiment and assembled to networks (Figure 1b and Figures S1–S10) may inspire expansion of hypothesis-driven research in the field.

For example, a key facet of the model regarding glaucomatous retinal neurodegeneration from our proteomics study may be impaired bioenergetics, which also stimulates other downstream or upstream stress-responsive signal transduction pathways (Figure 1b). Retinal neural networks have a very high-energy demand [45,46,47]. Glycolysis, oxidative phosphorylation, Ca^2+^ homeostasis, and neuronal excitability thus play critical roles in maintaining the functionality of the retinal cells [48]. In response to increased IOP, the mitochondrial architecture can be severely impacted, leading to a crisis in bioenergetics with elevated oxidative stress, with the eventual loss of RGCs causing visual impairment [46]. One of the predominant pathologies of glaucoma is associated with interference of energy homeostasis by making metabolic energy substrates unavailable [49]. Eventually, this disruption leads to amplified oxidative stress and mitochondrial dysfunction, as the mitochondria also play significant roles in assimilating intrinsic and external signals to regulate cell cycle and cell fate [50,51]. In a recent study conducted in glaucomatous human stem cell differentiated retinal ganglion cells (hRGCs), flow cytometry and different (immunofluorescence, confocal, and electron) microscopy techniques have revealed a novel neuroprotective mechanism of homeostasis under OHT by which hRGCs maintain rapid mitochondrial biogenesis and degradation [52]. This observation was attributed to hRGCs’ high vulnerability toward mitochondrial dysfunction due to their structural and functional uniqueness.

With our bioinformatic analyses, we also aspired to dissect some of the signaling mechanisms in response to mitochondrial dysfunction in the chosen rat model of glaucoma. To this end, we show the protein interaction network of Figure 1b (linked by IPA^®^ to development disorder, ophthalmic disease, organismal injury, and cellular compromise) with canonical pathways overlay focusing on essential biological functions modulated by OHT. Protein ubiquitination involving the proteasome, heat-shock proteins, and crystallins is associated with the maintenance of cell morphology, uptake of nutrients, synaptogenesis, apoptosis, and deregulation of this pathway has been shown to play a role in ocular neurodegeneration [46,53]. The network also calls attention to OHT-induced inhibition of the AKT (serine/threonine kinase family) signaling for involvement in the estrogen receptor signaling pathway [54,55,56,57]. In addition, the protein interaction network of Figure 2a indicated OHT’s triggering of the S100 and chaperone-mediated autophagy signaling pathways that participate in regulating cell growth, repair, and development.

Visual cycle proteins were also shown to be impacted by OHT (Figure 1b). In the visual cycle, the light-sensitive chromophore isomer of vitamin A is continually recycled through a biochemical pathway involving the retinal pigment epithelium to supply photoreceptors (rod and cone cells) and ensure proper visual function. Deregulation of this cycle leads to impaired phototransduction necessary to convert light signal to neural signal [58]. In Figure 1b, IPA^®^ included a network of NAD/NADP oxidoreductases and aldehyde reductase, which are involved in the visual cycle. Retinal dehydrogenases belong to the NADP oxidoreductase family that catalyzes the synthesis of the main active metabolite of the visual cycle’s vitamin A, the all-trans-retinoic acid [58,59]. Therefore, the observed upregulation of these proteins by OHT may indicate compensatory mechanisms due to impaired phototransduction in photoreceptors. However, structural damage of photoreceptors in glaucoma is still controversial based on a spectral-domain optical coherence tomography study [60]. On the other hand, neural retina apoptosis, axon damage, and synapse loss have been shown to affect RGCs, amacrine cells, and cone photoreceptor cells in another investigation using an acute mouse OHT model and immunofluorescence and terminal deoxynucleotidyl transferase deoxyuridine triphosphate nick end labeling (TUNEL) staining [61]. 

Deposition of the acquired raw data to make them freely available for reanalyses (see data availability statement below) could also guide studies focused on the identification of disease biomarkers and/or potential therapeutic targets regarding the impact of elevated IOP on the retina proteome through follow-up targeted approaches. In this context and as an example, we relied on the PRM method [34] that focused on the relative quantification of selected proteins found to be significantly dysregulated by OHT (Figure 2b). Our chosen mechanistic focus for this selection was retinal function, protein homeostasis, and bioenergetics based on the results of the discovery-driven study and the subsequent bioinformatic analyses thereof. Mitochondrial dysfunction, S100 family signaling, and chaperon-mediated autophagy signaling were indeed among the highly regulated canonical pathways implicated in the interaction network assembled from the ten selected proteins (Figure 2a). Targeted proteomics is also more accurate in assessing fold changes in protein expressions than the shotgun approach used for discovery-driven “global” proteomics [62]. Like with a rat model of diabetic retinopathy [63], confirmation of up- and downregulations of our selected ten proteins in their interaction network also offers their potential use as a focused panel of preclinical biomarkers for investigational therapy development against retinal neurodegeneration through the hypertonic saline injection model of experimental glaucoma in rats. Although we could label many proteins in our networks (Figure 1b, Figure 2a and Figures S1–S10) with their associated retinal cell types using IPA^®^’s cells and tissue overlay option, enrichment of target cells has been recommended for proteomics studies focusing on cell-specific subproteomes of the retina [64].

In conclusion, our label-free proteomics study identified over 200 retina proteins significantly affected by OHT in the hypertonic saline injection rat model of glaucoma. We revealed various aspects of glaucomatous pathophysiology through the organization of the findings into protein interaction networks and by pathway analyses. Focusing on retinal neurodegeneration as a characteristic process of the disease that leads to diminished or loss of vision, OHT-induced alterations in the expression of selected proteins in this context were verified by targeted proteomics. Acquired raw data were shared through deposition to the ProteomeXchange Consortium [31] by the PRIDE partner repository (assigned dataset identifier: PXD042729 and 10.6019/PXD042729) making a retina proteomics dataset on an induced OHT model of glaucoma available for the first time.

## 4. Materials and Methods

### 4.1. Chemicals and Reagents

Chemicals were purchased from Millipore Sigma (St. Louis, MO, USA). Sequencing-grade trypsin was from Promega (Madison, WI, USA). Chromatographic solvents were Optima^®^ LC/MS grade and supplied by Thermo Fisher Scientific (Waltham, MA, USA).

### 4.2. Animals 

Retired breeder BN male rats (8–10 months old, weighing 350–400 g) were purchased from Charles Rivers Laboratories (Wilmington, DE, USA). All performed procedures involving these animals conformed to the ARVO Statement for the Use of Animals in Ophthalmic and Vision Research and were approved by the Institutional Animal Care and Use Committee at the University of North Texas Health Science Center.

### 4.3. Induction of OHT and IOP Measurement

OHT was induced unilaterally in anesthetized animals (60 mg/kg body weight ketamine and 10 mg/kg body weight xylazine, administered intraperitoneally) by episcleral injection of 1.8 M saline, as reported before [27,28,65]. IOP was measured in awake rats with a hand-held Tono-Pen XL tonometer (Colonial Medical Supply, Londonderry, NH, USA) longitudinally, twice weekly throughout the duration of the study, between 9:00 AM and 10 AM to minimize diurnal variability in IOP. Pressure readings (6 to 10) were recorded and averaged for one IOP measurement. Baseline IOPs were recorded in each animal. The cumulative baseline average was 15 ± 1 mmHg (mean ± SD). IOP elevation was achieved after approximately 7–10 days post-injection in about 60% of animals. Once the IOP increased at least 25% over the corresponding baseline value, animals were assigned to the OHT group (n = 6). In this group, a sustained cumulative net IOP increase (6 ± 2 mmHg, mean ± SD) was achieved over the 3-week follow-up period, similar to our earlier report [65]. Age-matched animals that received no episcleral saline injections were included as controls (n = 4).

### 4.4. Retina Collection

Three weeks after achieving OHT [65], animals in each group were euthanized (60 mg/kg body weight ketamine and 10 mg/kg body weight xylazine, administered intraperitoneally), followed by decapitation. The eyes were then enucleated immediately, and the retinae were collected and stored at −80 °C until processing.

### 4.5. Discovery-Driven Label-Free Retina Proteomics

Protein extraction from the retinae, reduction, alkylation, trypsin digestion, and sample cleanup were conducted according to our previously reported procedures [65,66,67]. Samples (1 µg/µL protein content) were reconstituted in 5% *v*/*v* aqueous acetonitrile containing 0.1% *v*/*v* formic acid. Data-dependent LC-ESI-MS/MS was run on an LTQ Orbitrap Velos Pro mass spectrometer connected to EASY nLC-1000 systems and fitted with an EASY-Spray source (Thermo Fisher Scientific, San Jose, CA, USA). A source voltage of 2.2 kV and ion-transfer tube temperature of 275 °C were used. Nanoflow separations were performed on a 15 cm × 75 μm i.d. EASY-Spray column packed with 3 µm PepMap C18 particles. Peptides were eluted at 300 nL/min flow rate with a 2 h binary solvent gradient: solvent A and solvent B were water and acetonitrile, respectively, and each contained 0.1% (*v*/*v*) formic acid. Five µL of the sample solution was injected while maintaining constant column pressure at 600 bar during 20 min of column equilibration at 5% B. Then, the tryptic peptides in the sample were eluted using the following gradient: (i) 5 min isocratic at 5% B; (ii) linear program to 40% B over 90 min, and then (iii) isocratic at 40% B for 5 min; (iv) to 90% B over 5 min; (v) isocratic at 90% B for 5 min; and (vi) resetting to 5% B in 20 min. During elution, full-scan mass spectra (MS) were acquired with a nominal resolution of 60000 (at *m*/*z* 400) in the Orbitrap, and up to 20 MS-dependent tandem mass spectra (MS/MS) were obtained in the ion trap. Each full MS/MS spectrum was acquired using collision-induced dissociation (CID) of only multiply charged ions (z ≥ 2). After the selection of the ion to be fragmented, dynamic exclusion was set for 60 s. Two technical repeats were analyzed for each sample.

MS/MS spectra were searched against the UniProt protein sequence database (species: *Rattus norvegicus*, 2022; 36,254 entries) using the Mascot search algorithm (version 2.6.2, Matrix Science, Boston, MA, USA) within the Proteome Discoverer (version 2.4;Thermo Fisher Scientific, San Jose, CA, USA) software. Parent ion tolerance of 25 ppm, fragment ion mass tolerance of 0.80 Da, and one missed cleavage were set as search filters. Fixed and variable modifications included carbamidomethylation of cysteine and oxidation of methionine, respectively. Search results were validated to meet strong criteria of protein identifications using the Peptide Prophet [68] and Protein Prophet [69] algorithms requiring >95% and >99% probabilities, respectively, and at least two identified unique peptides for each protein using the Scaffold software (version 5.0.1; Proteome Software Inc., Portland, OR, USA).

Our LFQ relied on spectral counting [66,67] built into the Scaffold software, and *p* < 0.05 was considered significantly different using Fisher’s exact tests [32] upon comparing protein abundances between the OHT and normotensive retinae. Benjamini–Hochberg corrections were applied for multiple testing [33], and ≥2-fold difference in spectral counts between the two study groups was set as a threshold of significant change in protein abundances. Missing values, if any, were handled using Scaffold’s default method and settings. 

The identified IOP-regulated proteins were submitted to Ingenuity Pathway Analysis^®^ (IPA^®^, QIAGEN, Redwood City, CA, USA) to derive bioinformatics annotations along with potential protein interaction networks, as well as associated biological functions and processes. Overlaps of *p*-values were reported from IPA^®^’s calculations using the right-tailed Fisher’s exact test [70]. Z-scores were generated for regulated functions, together with their predicted signaling patterns, through the MAP tool built into IPA^®^.

### 4.6. Targeted Proteomics 

Chromatographic and nESI conditions were identical to those described for the discovery-driven shotgun method above. Peptide ID files with .mzid extensions were exported from Scaffold (software specified in the previous section) and used along with the spectral database of the National Institute of Standards and Technology [71] to generate a library of spectra in Skyline (version 21.1.0.146, MacCoss Lab Software, University of Washington, Seattle, WA, USA) [54]. For the selected proteins, sequences, spectra, and fragmentation tables of the tryptic peptides were also obtained from label-free shotgun data using the Scaffold. Peptides for PRM were chosen with criteria including 100% identification probability, no missed cleavage and posttranslational modification, doubly charged precursor ions (z = 2), and listed with retention time windows in Table S8. Full MS/MS scans were acquired using 2.0 Th isolation width, CID at 35% normalized collision energy, and 30 ms activation time. The Orbitrap’s nominal mass resolution was set to 15,000 (at *m*/*z* 400). 

PRM raw data files were imported into Skyline [72], and the precursor and product ion chromatograms were extracted and analyzed using its built-in PRM mode. Retention time scheduling was also performed from these raw data files. Precursor charge was set to 2, and product ion charges were 1 and 2 with b- and y-type fragment ions considered. Ion match tolerance was 0.5 Da to search for product ions. Relative abundances of the peptides were used for relative quantitation. To obtain fold changes, all the technical replicates of all samples for each peptide (Table S8) were averaged first, and then the treated samples were compared to controls. For validation, the peptide sequence was confirmed by the acquired MS/MS scan from b- and y-type sequence ions marked according to the nomenclature by Roepstorff and Fohlman [73]. Group comparisons were performed using the transitions reported by Skyline’s descriptive statistics calculated for each group and all outcomes. Differences of mean values between experimental groups were assessed by unpaired *t*-tests. In all comparisons associated with targeted proteomics, *p* < 0.05 was considered statistically significant.

## Figures and Tables

**Table 1:** (**a**) Molecular and cellular processes, as well as (**b**) diseases and physiological functions represented by the significantly affected retina proteins of retired-breeder male BN rats after the elevation of their IOP compared to normotensive age-matched controls.

(**a**)		
**Represented Process**	**Number of Associated Molecules**	***p*** **-Value of Overlap**
Cellular function and maintenance	19	1.96 × 10^−6^–4.76 × 10^−2^
Cellular assembly and organization	18	1.96 × 10^−6^–4.76 × 10^−2^
Cellular compromise	15	4.99 × 10^−5^–4.24 × 10^−2^
Cell death and survival	14	1.93 × 10^−5^–4.76 × 10^−2^
Cellular movement	13	1.96 × 10^−6^–4.99 × 10^−2^
(**b**)		
**Associated Disease**	**Number of Associated Molecules**	***p*** **-Value of Overlap**
Organismal injury and abnormalities	31	1.46 × 10^−6^–4.33 × 10^−2^
Neurological disease	27	1.38 × 10^−4^–4.33 × 10^−2^
Metabolic disease	9	3.38 × 10^−4^–3.48 × 10^−2^
Ophthalmic disease	8	4.00 × 10^−6^–3.94 × 10^−2^
Endocrine system disorder	4	3.38 × 10^−5^–3.38 × 10^−5^

## Data Availability

The mass spectrometry proteomics data have been deposited to the ProteomeXchange Consortium via the PRIDE [31] partner repository with the dataset identifier PXD042729 and 10.6019/PXD042729.

## Supplementary Materials

The following supporting information can be downloaded at: https://www.mdpi.com/article/10.3390/ijms241612592/s1.
