# Supplementary material for: Proteomics-Based Identification of Retinal Protein Networks Impacted by Elevated Intraocular Pressure in the Hypertonic Saline Injection Model of Experimental Glaucoma
Source: Int J Mol Sci. 2023 Aug 9;24(16):12592. doi: 10.3390/ijms241612592 (PMC10454042; doi:10.3390/ijms241612592)
Supplement: Supplementary file 1 [file ijms-24-12592-s001.zip › FiguresS4.pdf]

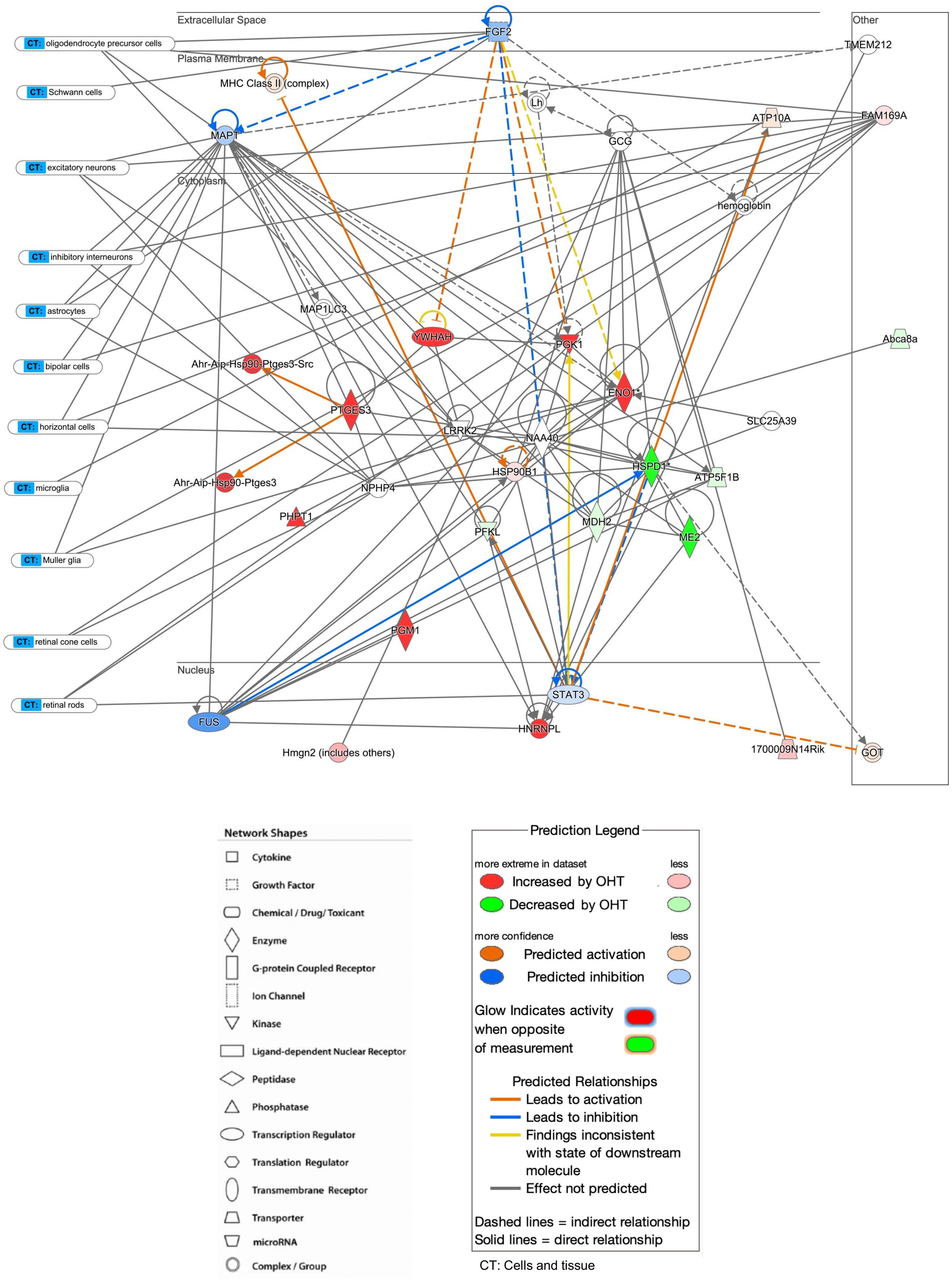

Figure S4. IPA® network linked to energy production, nucleic acid metabolism, small molecule biochemistry.
