# Supplementary figures and images for: Proteomics-Based Identification of Retinal Protein Networks Impacted by Elevated Intraocular Pressure in the Hypertonic Saline Injection Model of Experimental Glaucoma
Source: Int J Mol Sci. 2023 Aug 9;24(16):12592. doi: 10.3390/ijms241612592 (PMC10454042; doi:10.3390/ijms241612592)

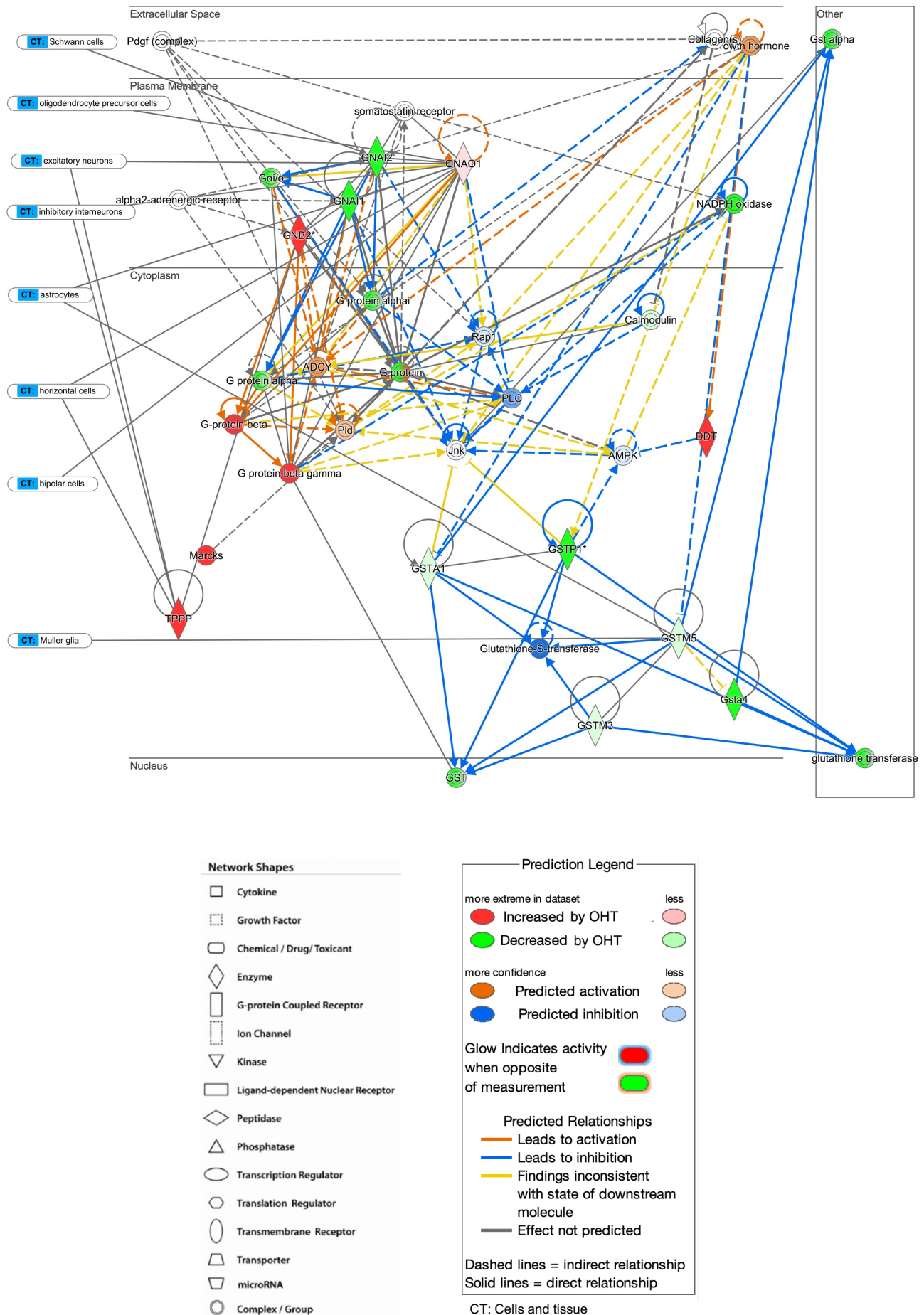

**Figure S6.** IPA® network linked to drug metabolism, glutathione depletion, protein synthesis.

Supplement: Supplementary file 1 [file ijms-24-12592-s001.zip › FiguresS6.pdf]

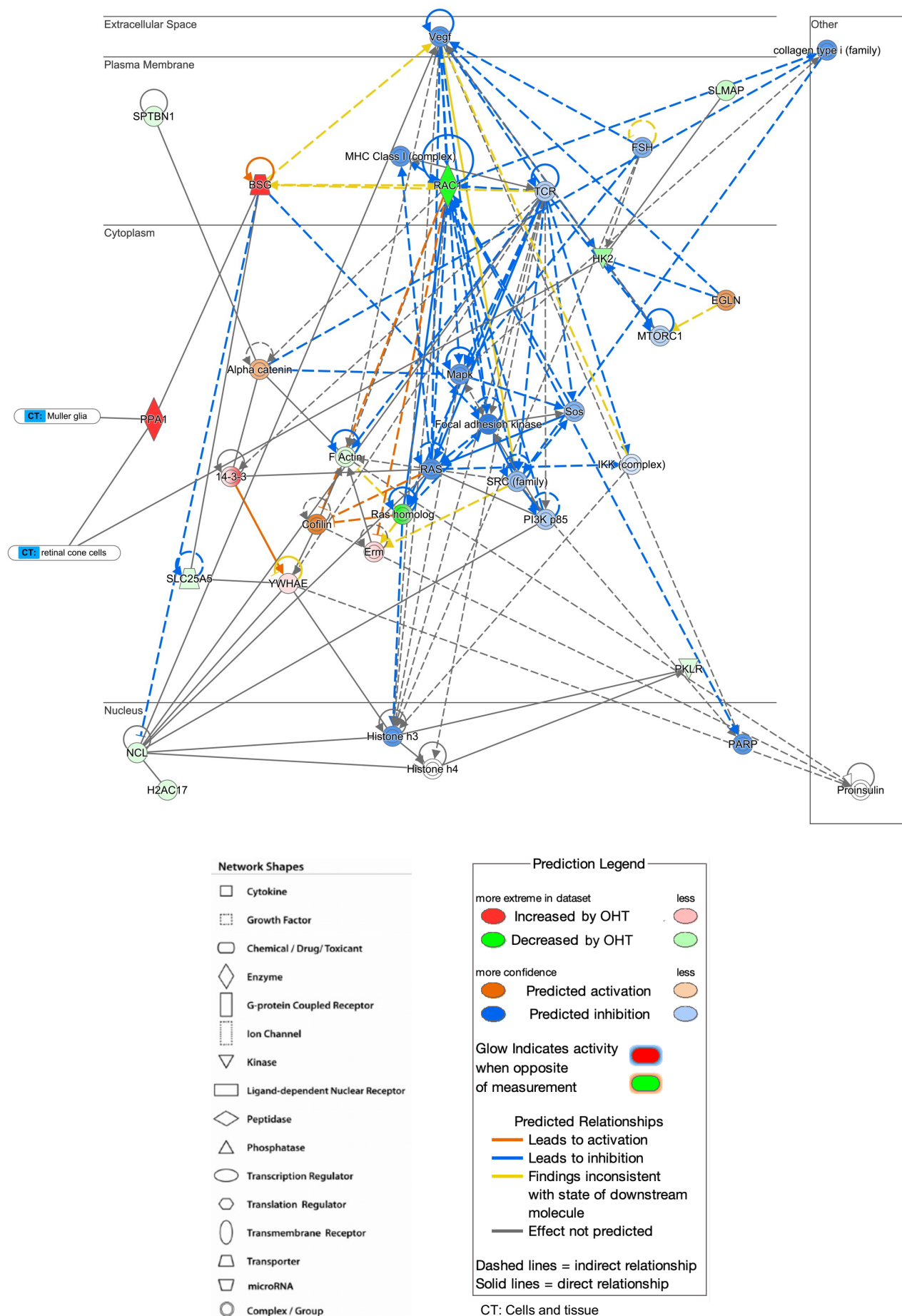

**Figure S7.** IPA® network linked to cancer, nucleic acid metabolism, small molecule biochemistry.

Supplement: Supplementary file 1 [file ijms-24-12592-s001.zip › FiguresS7.pdf]

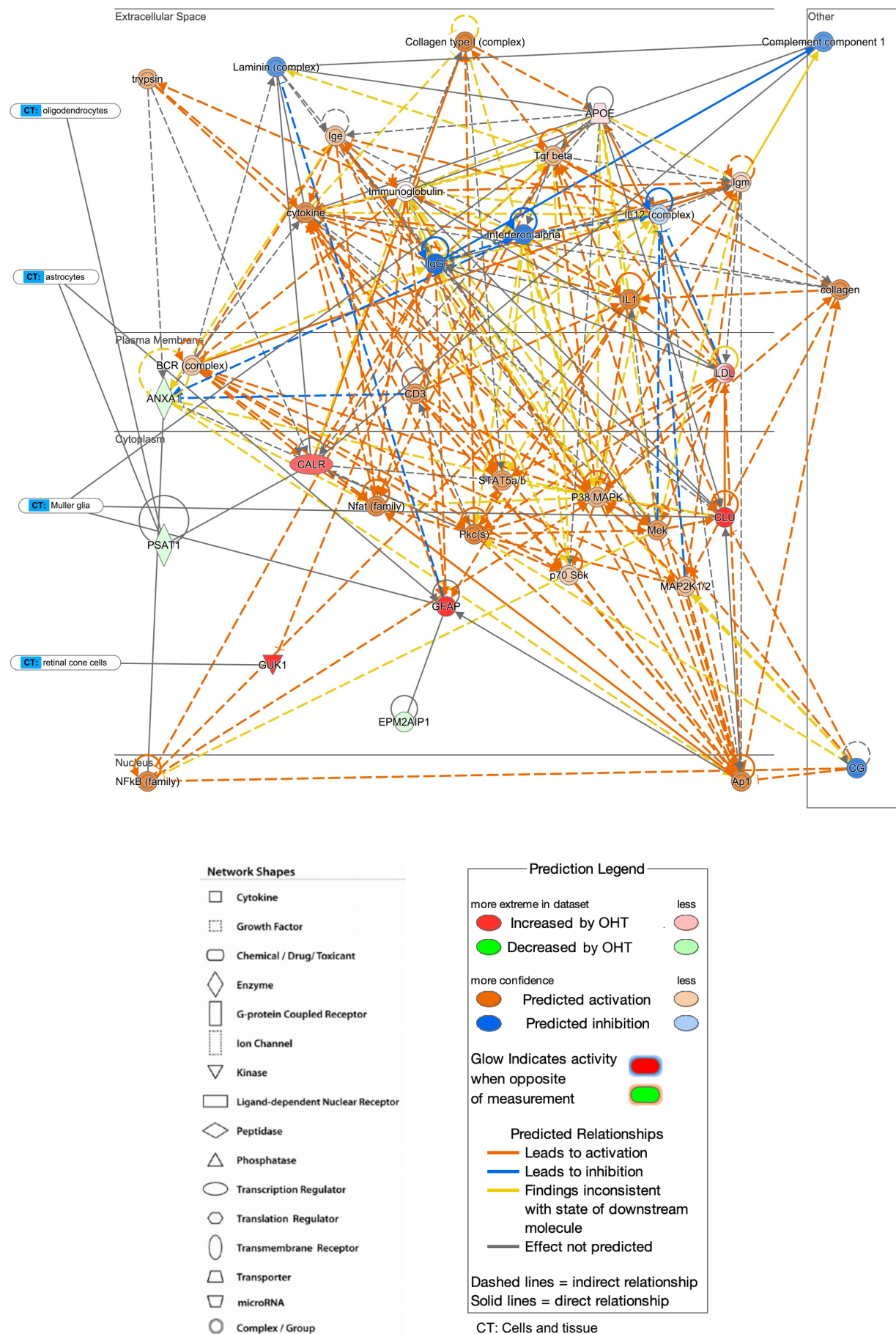

**Figure S9.** IPA® network linked to organ morphology and organismal development and function.

Supplement: Supplementary file 1 [file ijms-24-12592-s001.zip › FiguresS9.pdf]
